# Use of insulin pump therapy is associated with reduced hospital-days in the long-term: a real-world study of 48,756 pediatric patients with type 1 diabetes

**DOI:** 10.1007/s00431-020-03883-2

**Published:** 2020-12-01

**Authors:** Marie Auzanneau, Beate Karges, Andreas Neu, Thomas Kapellen, Stefan A. Wudy, Corinna Grasemann, Gabriele Krauch, Eva Maria Gerstl, Gerhard Däublin, Reinhard W. Holl

**Affiliations:** 1 Institute of Epidemiology and Medical Biometry, ZIBMT, University of Ulm, Albert-Einstein-Allee 41, D-89081 Ulm, Germany; 2 German Center for Diabetes Research (DZD), Neuherberg, Germany; 3 Division of Endocrinology and Diabetes, Medical Faculty, RWTH Aachen University, Aachen, Germany; 4 University Children’s Hospital Tübingen, Tübingen, Germany; 5 Department of Women and Child Health, Hospital for Children and Adolescents, University of Leipzig, Leipzig, Germany; 6 Division of Pediatric Endocrinology and Diabetology, Center of Child and Adolescent Medicine, Justus Liebig University, Giessen, Germany; 7 St. Josef-Hospital, Klinikum der Ruhr-Universität, Bochum, Germany; 8 Division of Pediatric Endocrinology and Diabetology, Center of Child and Adolescent Medicine, University Medicine, Mannheim, Germany; 9 Children’s Hospital Passau, Passau, Germany; 10 Children’s Hospital Aurich, Aurich, Germany

**Keywords:** Hospitalization, Hospital days, Insulin pump therapy, Children, Type 1 diabetes

## Abstract

In pediatric diabetes, insulin pump therapy is associated with less acute complications but inpatient pump education may lead to more hospital days. We investigated the number of hospital days associated with pump vs. injection therapy between 2009 and 2018 in 48,756 patients with type 1 diabetes < 20 years of age from the German Diabetes Prospective Follow-up Registry (DPV). Analyses were performed separately for hospitalizations at diagnosis (hierarchical linear models adjusted for sex, age, and migration), and for hospitalizations in the subsequent course of the disease (hierarchical Poisson models stratified by sex, age, migration, and therapy switch). At diagnosis, the length of hospital stay was longer with pump therapy than with injection therapy (mean estimate with 95% CI: 13.6 [13.3–13.9] days vs. 12.8 [12.5–13.1] days, *P* < 0.0001), whereas during the whole follow-up beyond diagnosis, the number of hospital days per person-year (/PY) was higher with injection therapy than with pump therapy (4.4 [4.1–4.8] vs. 3.9 [3.6–4.2] days/PY), especially for children under 5 years of age (4.9 [4.4–5.6] vs. 3.5 [3.1–3.9] days/PY).

*Conclusions:* Even in countries with hospitalizations at diabetes diagnosis of longer duration, the use of pump therapy is associated with a reduced number of hospital days in the long-term.

**Table:** 

**What is known:** *• In pediatric diabetes, insulin pump therapy is associated with better glycemic control and less acute complications compared with injection therapy.* *• However, pump therapy implies more costs and resources for education and management.* **What is new:** *• Even in countries where pump education is predominantly given in an inpatient setting*, *the use of pump therapy is associated with a reduced number of hospital days in the long-term.* *• Lower rates of hospitalization due to acute complications during the course of the disease counterbalance longer hospitalizations due to initial pump education*

**What is known:**

*• In pediatric diabetes, insulin pump therapy is associated with better glycemic control and less acute complications compared with injection therapy.*

*• However, pump therapy implies more costs and resources for education and management.*

**What is new:**

*• Even in countries where pump education is predominantly given in an inpatient setting*, *the use of pump therapy is associated with a reduced number of hospital days in the long-term.*

*• Lower rates of hospitalization due to acute complications during the course of the disease counterbalance longer hospitalizations due to initial pump education*

## Introduction

In children and adolescents with type 1 diabetes, the use of diabetes technology has rapidly increased worldwide. For instance in the USA [1], in Nordic countries [2], and in Germany or Austria [3], the majority of patients aged < 15 years use insulin pump therapy. In agreement with the growing evidence that insulin pump therapy is associated with better glycemic control [4], less acute complications [5], and lower cardiovascular mortality [6] compared to injection therapy, most health care providers judge insulin pump therapy as safe and effective. In addition, preadolescent children and caregivers report substantial psychosocial benefits and improved quality of life in relation with the use of insulin pumps [7].

However, pump therapy remains more expensive than injection therapy [8]. Pump therapy implies not only more material costs but also more resources and time spent for education and management [8]. Currently, practices of insulin pump initiation, education, and training vary a lot around the world [9, 10]. For instance, in New Zealand, both out- and inpatient approaches of pump initiation and training have been described [9]. In the USA, pump training typically occurs several months after diagnosis and is done completely outpatient. By contrast, in other countries like in Sweden, Austria, or Germany, pump initiation and education mostly occur in an inpatient setting [10, 11]. German guidelines for instance particularly recommend pump therapy (beside many other criteria) for all newborns, infants, and preschoolers, and for these children, pump therapy is mostly initiated during a one or two weeks hospitalization immediately after diagnosis [5, 11, 12].

Inpatient management and education for diabetes technology may lead to an increased number of hospital days for children using pump therapy. On the other hand, studies have shown that the use of pump therapy is associated with a lower number of hospital stays due to acute complications (diabetic ketoacidosis [DKA] or severe hypoglycemia) compared to injection therapy [5]. Therefore, in countries with predominantly inpatient diabetes education, there is still an uncertainty whether the use of pump therapy is associated with a lower or with a higher number of hospital days compared with injection therapy in the long-term.

In the present study, we investigated the number of hospital days per person-year between 2009 and 2018 associated with the use of insulin pump therapy vs. injection therapy in 48,756 pediatric patients with type 1 diabetes in Germany.

## Material and methods

### Study population

For this population-based cohort study, we used data from the multicenter Diabetes Prospective Follow-up (DPV) Initiative based at the University of Ulm, Germany. Since 1995, all hospitals and practices participating in the DPV registry, mainly located in Germany and Austria, prospectively document clinical and demographic data of patients with any type of diabetes. The documentation is done both manually and automatically (download from clinic information systems) into the predefined database DPV, a diabetes-specific electronic health record. Every 6 months, the collected data is transmitted after verification in anonymous form to the University of Ulm. Subsequently, the university of Ulm reports implausible data back to centers, and performs central analysis and quality assurance. The Ethics Committee of the Medical Faculty of the University of Ulm, as well as the local review boards of participating centers, approved both data collection and analysis of anonymized data from the DPV database.

Since data documentation in the DPV registry is closely controlled, many variables (e.g., sex, age, diabetes duration, or migration status) have no missing values. Other data, like hospital days, are not completely documented. However, hospital days in the DPV database are extracted from administrative data collected by the diabetes center, relevant for reimbursement, and as such their documentation is particularly controlled by the hospital and by external controllers, for example, by the health insurance companies.

As of March 2019, data of 556,021 patients from 485 diabetes centers were documented in the database. For this analysis, we included all patients with a clinical diagnosis of type 1 diabetes at the age of six months or later, aged < 20 years, living in Germany, documented between January 1, 2009, and December 31, 2018, with at least two visits (outpatient or inpatient), and available documentation of insulin pump therapy or insulin injection therapy. Hospital stays longer than three weeks were set to 21 days, considering that other causes, not related to diabetes or diabetes therapy may have led to such exceptional length of stay. Patients from countries outside of Germany were excluded due to differences between national health systems.

### Explanatory variables

In order to adjust for differences between local laboratories, we standardized individual HbA1c values to the Diabetes Control and Complications Trial reference of 4.05–6.05%, using the “multiple-of-the-mean” transformation method [13]. We transformed BMI (kg/m^2^) values as standard deviation score (SDS) to adjust for age and gender, using a German reference population [14] and the LMS method [15]. Age was categorized in four groups: 0.5 to < 5 years, 5 to < 10 years, 10 to < 15 years, and 15 to < 20 years. Migration background was defined as birth of the patient himself or at least one of his parents outside of Germany.

### Statistical analysis

We separately investigated the number of hospital days associated with pump or injection therapy for the period of diagnosis and during the subsequent course of the disease.

For the period of diagnosis, we performed hierarchical linear regression models adjusting for sex, age group, and migration background, and presented results as number of hospital days at diagnosis with 95% confidence interval (95% CI). As not all patients are hospitalized on the day of diagnosis, we used a time-interval of 9 days before and after the date of diagnosis to identify hospitalizations related to the onset of diabetes. If the day of admission to the hospital fell within this period, then the entire length of the hospital day was considered to represent the hospitalization at onset of diabetes (i.e., at diagnosis). Patients hospitalized with reason “onset” 10 days or more after diagnosis were assumed to have been identified as patient with presymptomatic diabetes by previous screening. We excluded those patients (*n* = 990) as they are generally more informed about diabetes and usually do not experience the typical sudden manifestation of the disease [16].

For the investigation of hospitalization during the course of the disease (hospital admission 10 days or more after diabetes diagnosis), we performed hierarchical Poisson regression analyses taking into consideration for each patient the individual cumulative time at risk spent with each treatment modality between 2009 and 2018. Results are given as number of hospital days per person-year (/PY) with 95% CI. Time at risk and number of hospital days, both related with either injection therapy or pump therapy, were summed up, including within patients with therapy switch. Other parameters were aggregated as median.

For hospital stays with a switch of therapy, we divided the number of hospital days half-and-half into days with pump and days with injection therapy. We also performed a sensitivity analysis to test other ways of dividing the days allocated to the previous and to the new therapy (30–70%, or 20–80% respectively), considering the possibility of a planned switch, which rather occurs at the beginning of the hospital stay, or of a delayed switch, which is decided during the hospitalization period. If hospital stay with a switch of therapy was the last documented visit, we only took into account half the number of days (or 30% and 20% respectively in the sensitivity analysis), and allocated them to the therapy modality of the beginning of the hospital stay, as hospital days of the new treatment could not be related to a sufficient observation time with the new therapy.

Regression models were stratified by sex, age group, and migration background. Additionally, we performed the Poisson regression analysis in the subgroup of patients with at least one therapy switch, assuming that patients who have used pump therapy continuously and those who have used injection therapy continuously may have different baseline characteristic associated with both the choice of the therapy and their hospitalization risk (confounding by indication). We analyzed for each patient the cumulative time spent with injection therapy and the cumulative time spent with pump therapy separately. Thus, comparisons were conducted within the same patients, so that baseline characteristics were identical. Moreover, we considered the direction of the switch and repeated the analysis within patients who switched at least once from injection to pump, from pump to injection, and in both directions.

In a second step, we investigated with the same Poisson regression models the number of hospital days per person-year associated with pump or injection therapy for the most frequent admission causes documented in the DPV registry, namely: DKA, severe hypoglycemia, and diabetes education.

We used random intercept models (variable “diabetes center” included as a random effect, with Cholesky parameterization of an unstructured covariance matrix) to take into account the possible hierarchical structure of the data (patients from the same center are supposed to have more similar characteristics than patients from two different centers). All regression analyses were complete case analyses. We present descriptive data as median (interquartile range, IQR) for continuous variables or percentage for categorical variables. Wilcoxon test was used for comparison of continuous variables and *X*^2^ test was used to compare variables with binomial distribution. Due to the large size of the study population, the level of significance of two-sided tests was set at *P* < 0.01, as generally recommended in biostatistical textbooks. All statistical analyses were performed with SAS 9.4 (SAS Institute, Cary, NC, USA).

## Results

### Characteristics of the study population

Of 84,344 patients with a clinical diagnosis of type 1 diabetes at the age of 6 months or later aged < 20 years from the DPV registry, 79,241 were living in Germany; 55,234 were documented between January 1, 2009, and December 31, 2018; 49,427 had at least two visits (outpatient or inpatient); and for 48,756 (our final study population), the documentation of insulin pump therapy or insulin injection therapy was available.

### Analysis for the period of diagnosis

Among all patients included, 22,434 children had data documented during the first 9 days around diagnosis (Table 1). At diagnosis, the large majority of children were treated with insulin injections (*n* = 18,080; 80.6%). Of those children, 3,092 (17.1%) used conventional insulin therapy (CT) (1–3 daily injection time points), and 14,988 (82.9%) used intensified insulin therapy (ICT) / basis bolus therapy (> 3 daily injection time points). Children who received pump therapy at diagnosis were in median younger than those using injection therapy (3.7 years vs. 10.9 years, *P* < 0.0001, Table 1). At diagnosis, the length of hospital stay was longer with pump therapy, even after adjustment for sex, age group, and migration background (13.6 [95%-CI: 13.3–13.9] vs. 12.8 [12.5–13.1] days, *P* < 0.0001, Table 2).

**Table 1:** Characteristics of the study population in the period of diagnosis

	All patients (*n* = 22,434)	Injection therapy (*n* = 18,080)	Pump therapy (*n* = 4,354)	*P* values^a^
Girls, *n* (%)	10,185 (45.4)	8,073 (44.7)	2,111 (48.5)	< 0.0001
Age, years (median, IQR)	9.7 (6.0–12.9)	10.9 (8.1–13.5)	3.7 (2.4–5.3)	< 0.0001
Migration background, *n* (%)	4,980 (22.2)	3,978 (22.0)	1,002 (23.0)	n.s. (0.15)
BMI SDS (median, IQR)	0.03 (− 0.56 to 0.70)	− 0.03 (− 0.63 to 0.66)	0.29 (− 0.21 to 0.86)	< 0.0001
HbA1c, % (median, IQR)	11.0 (9.5–12.6)	11.2 (9.7–12.9)	10.1 (8.9–11.5)	< 0.0001

*IQR* interquartile range. Migration background is defined as birth of the patient himself or at least one of his parents outside of Germany. *BMI SDS* standard deviation score of body mass index (kg/m^2^)

^a^Comparison between patients with pump or injection therapy using Wilcoxon test for continuous variables and *X*^2^ test for variables with binomial distribution

**Table 2:** Number of hospital days associated with injection vs. pump therapy in the period of diagnosis

	Injection therapy (*n* = 18,080)	Pump therapy (*n* = 4,354)	*P* values
Number of hospital days at diabetes onset (95% CI) ^a^			
Unadjusted	12.7 (12.4–12.9)	14.0 (13.7–14.3)	< 0.0001
Adjusted for sex, age group, and migration background	12.8 (12.5–13.1)	13.6 (13.3–13.9)	< 0.0001

^a^Mean estimates from linear regression models, with diabetes center as random effect

Migration background is defined as birth of the patient himself or at least one of his parents outside of Germany

### Analysis during the subsequent course of the disease

**Fig. 1:**
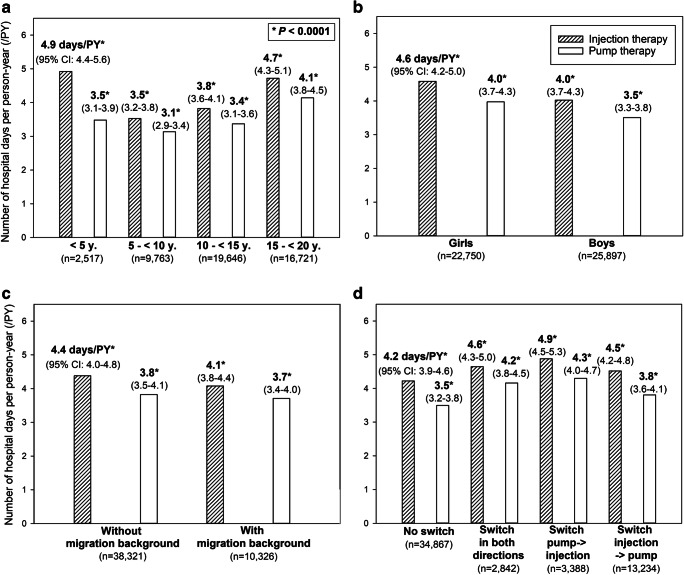
Number of hospital days associated with injection vs. pump therapy during the subsequent course of diabetes. Legend: mean estimates of Poisson regression models with 95%, CI. All comparisons between injection therapy and pump therapy groups are significant (*P* < 0.0001)

Of 48,647 patients with data documented beyond the period of diagnosis, 24,408 (50.2%) used injection therapy continuously (2007 on CT and 22,401 on ICT), whereas 10,459 (21.5%) used only pump therapy (Table 3). Among 13,780 (28.3%) patients who switched between both therapy options, 13,234 switched at least once from injection to pump, 3,388 switched at least once from pump to injection, and 2,842 patients switched in both directions (Fig. 1d).

**Table 3:** Characteristics of the study population during the subsequent course of the disease

	All patients (*n* = 48,647)	Patients without therapy switch (*n* = 34,867)			Patients with therapy switch (*n* = 13,780)	*P* values^b^
		Injection therapy continuously (*n* = 24,408)	Pump therapy continuously (*n* = 10,459)	*P* values^a^		
Girls, *n* (%)	22,750 (46.8)	10,364 (42.5)	5,222 (49.9)	< 0.0001	7,164 (52.0)	< 0.0001
Age, years (median, IQR)	13.5 (9.9–15.8)	14.5 (11.8–16.5)	9.5 (5.8–15.1)	< 0.0001	12.8 (10.2–14.8)	< 0.0001
Age group, *n* (%)						
0.5–< 5 years	2,517 (5.2)	271 (1.1)	1,925 (18.4)	< 0.0001	321 (2.3)	< 0.0001
5–< 10 years	9763 (20.1)	3,376 (13.8)	3,505 (33.5)	< 0.0001	2,882 (20.9)	0.003
10–< 15 years	19,646 (40.4)	9,940 (40.7)	2,340 (22.4)	< 0.0001	7,366 (53.5)	< 0.0001
15–< 20 years	16,721 (34.4)	10,821 (44.3)	2,689 (25.7)	< 0.0001	3,211 (23.3)	< 0.0001
Age at onset, years (median, IQR)	8.5 (4.9–11.9)	10.4 (7.0–13.2)	4.4 (2.6–7.0)	< 0.0001	8.3 (5.4–11.0)	< 0.0001
Diabetes duration, years (median, IQR)	3.0 (1.4–6.1)	2.5 (1.1–5.7)	4.0 (1.7–7.8)	< 0.0001	3.3 (1.9–5.5)	< 0.0001
Migration background, *n* (%)	10,326 (21.2)	5,592 (22.9)	1,943 (18.6)	< 0.0001	2,791 (20.3)	0.001
BMI SD (median, IQR)	0.33 (− 0.22 to 0.89)	0.28 (− 0.29 to 0.87)	0.44 (− 0.08 to 0.96)	< 0.0001	0.32 (− 0.22 to 0.88)	n.s. (0.18)
HbA1c, % (median, IQR)	7.6 (6.9–8.4)	7.6 (6.8–8.5)	7.6 (7.0–8.3)	n.s. (0.47)	7.6 (7.0–8.3)	n.s. (0.80)

Unadjusted data. *IQR* interquartile range. Migration background is defined as birth of the patient himself or at least one of his parents outside of Germany. *BMI SDS* standard deviation score of body mass index (kg/m^2^)

^a^Comparison between patients using injection or pump therapy continuously, performed with Wilcoxon test for continuous variables and *X*^2^ test for variables with binomial distribution

^b^Comparison between patients with or without therapy switch, performed with Wilcoxon test for continuous variables and *X*^2^ test for variables with binomial distribution

Compared with individuals who used injection therapy continuously, those who used only pump therapy during the subsequent course of the disease were in median five years younger, had earlier diabetes onset, and less often a migration background (Table 3, all *P* < 0.0001). Among the children and adolescents who used both therapy modalities, there was a majority of girls (52.0% vs. 46.8% in the study population), and of patients aged 10 to < 15 years (53.5% vs. 40.4% in the study population) (Table 3). Median HbA1c was similar (7.6%) in all groups (Table 3).

Over the 10 years of follow-up beyond the period of diagnosis, the number of hospital days per person-year was higher with injection than with pump therapy (unadjusted mean estimate with 95% CI: 4.4 [4.1–4.8] vs. 3.9 [3.6–4.2] days/PY, *P* < 0.0001). Stratified by age group, sex, migration status, or therapy change (continuous use or switch), the results were similar, with a higher number of hospital days per person-year associated with injection therapy than with pump therapy (Fig. 1, all comparisons significant with *P* < 0.0001). The largest difference was observed for children under 5 years of age, with 4.9 [4.4–5.6] days/PY associated with injection therapy vs. 3.5 [3.1–3.9] days/PY associated with pump therapy, *P* < 0.0001 (Fig. 1a). Even in the 13,780 children and adolescents who switched at least once between both therapy modalities, the number of hospital days associated with injection therapy was higher than with pump therapy, whatever the direction of the switch (for all patients with therapy switch: 4.6 [4.3–5.0] vs. 3.9 [3.6–4.2] days/PY, *P* < 0.0001; results detailed by direction of the switch: Fig. 1d). However, if we consider that most hospital days in case of hospitalization with therapy switch should be allocated to the new therapy, the advantage of the pump therapy in terms of reduced hospital days was no longer evident for patients with therapy switch (sensitivity analysis, results non shown here).

**Fig. 2:**
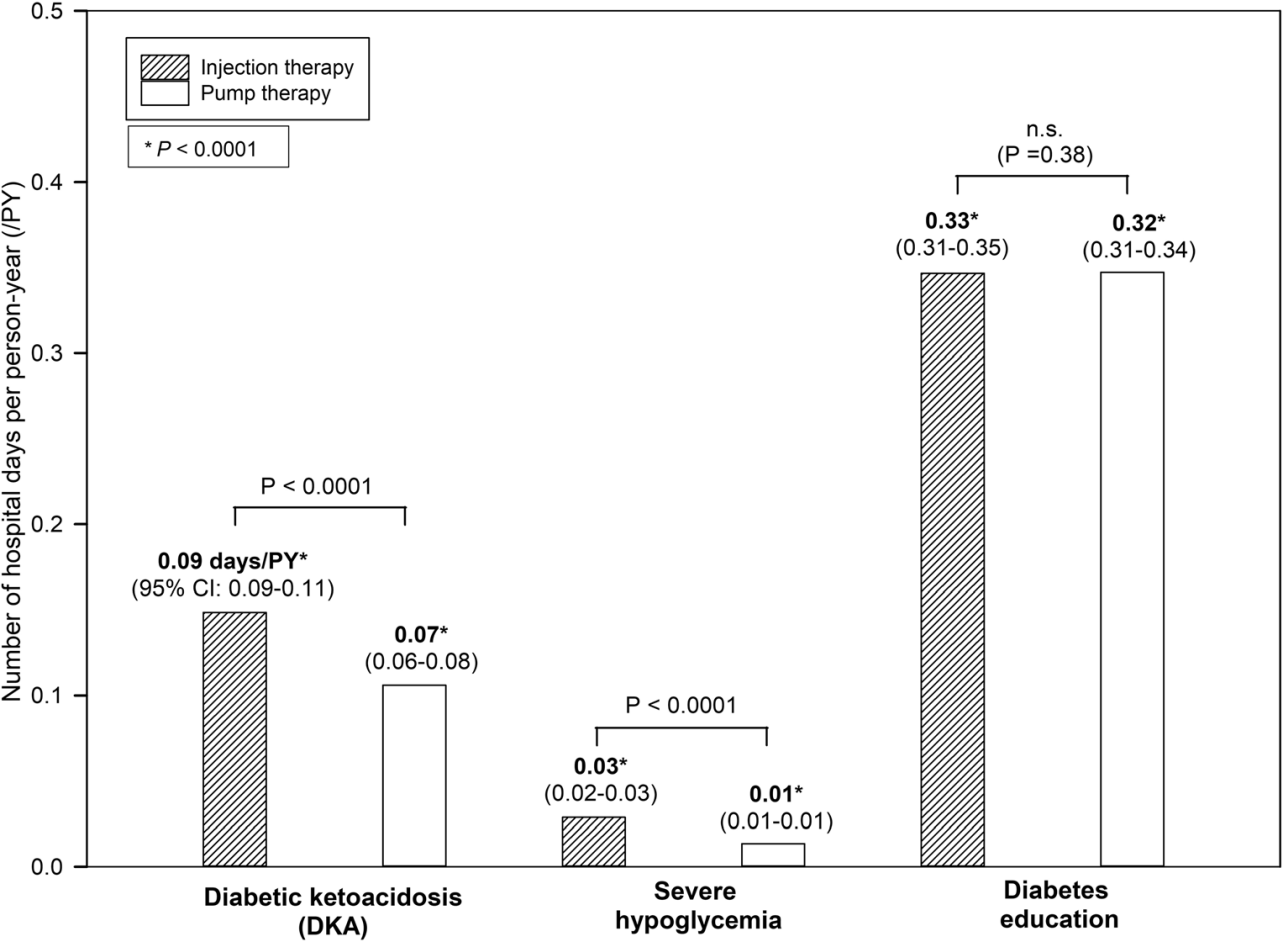
Number of hospital days associated with injection vs. pump therapy during the subsequent course of diabetes, by admission cause. Legend: mean estimates of Poisson regression models with 95%, CI

Regarding the most frequent admission causes, the numbers of hospital days per person-year due to diabetes education were similar with pump or injection therapy (Fig. 2). However, the numbers of days due to DKA or severe hypoglycemia associated with injection therapy were significantly higher than those associated with pump therapy (Fig. 2, both *P* < 0.0001).

## Discussion

This retrospective analysis of real-world data of 48,756 children and adolescents with type 1 diabetes over a period of 10 years in Germany showed different results for the number of hospital days associated with the type of therapy in the period of diagnosis and during the subsequent course of the disease. Whereas the length of the hospital stays was longer with pump therapy than with injection therapy in the period of diagnosis, the number of hospital days per person-year was in contrast lower with pump therapy during the subsequent course of the disease. Both results were robust, even after adjustment for sex, age group, and migration background for the period of diagnosis, or after stratification by age group, sex, migration status, or switch of therapy for the analysis during the subsequent course of the disease.

In the period of diagnosis, children with pump therapy were younger than those with injection therapy. This result is coherent with the German guidelines, which recommend pump therapy for all newborns, infants, and preschoolers [17]. For these children in Germany, insulin pump therapy is often initiated during the first hospitalization, immediately after the diagnosis of diabetes is made [12]. Compared to older children or adolescents, preschool children often have longer hospital stays [18] and they receive with their parents more education sessions [11]. Thus, the younger age of the pump users in the period of diagnosis may partly explain the longer hospital stays. However, the result was similar after adjustment for age groups, so that the additional time spent in hospital cannot be completely explained by the younger age.

Another plausible reason for longer hospital stays at diagnosis is the additional education and management for pump use compared to injection therapy. Indeed in Germany (in contrast to other countries, e.g., the USA), inpatient management of newly diagnosed diabetes including diabetes education is common, regardless of the choice of the therapy [11, 18]. However, diabetes education for parents of children using a pump therapy might require more time to learn how to manage the pump and adjust the dosages.

In the analysis during the subsequent course of the disease, we found - in contrast to the period of diagnosis that the number of hospital days per person-year was lower with insulin pump therapy than with injection therapy. The difference was small (less than one day per person-year), but estimated over a long period in a large population (201,442 patient-years), and robust even after different stratifications by age group, sex, migration background, or presence/absence of therapy switch, indicating that the results are independent of these characteristics. In particular, it is noteworthy that a similar result was obtained in patients who switched one or more times between insulin injections and pump, whatever the direction of the switch. In this sub-analysis, comparisons were performed within the same patients. Thus, even unmeasured demographic and psychosocial characteristics which are expected to influence the outcomes (e.g., family support, education level, deprivation, or socioeconomic status) were the same, and could not have influenced the findings. Nevertheless, a sensitivity analysis showed that the results for the patients with switch depend on the allocation of the hospital days for hospitalizations with therapy switch. If instead of 50%, 70% or 80% of these hospital days were allocated to the new therapy (i.e., for most of the patients, pump therapy), then the long-term advantage of the pump therapy was no longer evident for the patients with therapy switch, but if hospital stays with therapy switch are not taken into account (or with 50–50% of the days allocated to the previous and to the new therapy), then the use of pump therapy was associated with less hospital days, even in patients with therapy switch.

Our results indicate a reduced number of hospital days due to DKA or severe hypoglycemia in association with pump use. This is in line with the results of a large population-based cohort study, reporting an association between the use of insulin pump and lower rates of acute complications in patients under 20 years of age [5]. However, in this study and in our analysis, the education level or motivation of the family was not taken into account, so that a residual selection bias could not have been completely excluded. Older children who start pump therapy may come more frequently from higher motivated families, which can be a factor for less complications and less hospitalizations. In the present analysis, pump use was associated with a reduced number of hospital days, including in the subgroup of patients with migration background. However, a selection bias based on the motivation of the family for the use of diabetes technology is also possible in the subgroup of patients with migration background.

We found longer hospitalizations at diagnosis for pump users, but reduced hospital days in the long-term. Similarly, a pragmatic randomized controlled trial conducted in patients under 15 years of age in the UK concluded that insulin pump therapy is not cost-effective during the first year after diagnosis (including higher costs related to inpatient stays) [19]. However, a systematic review indicated that the higher lifetime direct costs associated with the pump therapy were partially offset by cost-savings from reduced diabetes-related complications, and that insulin pump therapy was cost-effective for patients with poor glycemic control or problematic hypoglycemia with injections [20].

The major strength of this study is the use of real-world data of a large population of children and adolescents with type 1 diabetes, documented over 10 years in a nationwide registry. The DPV database covers more than 90% of all pediatric patients with type 1 in Germany [3], and thus, the data used can be considered as representative. Although a risk of residual selection bias cannot completely be excluded in observational studies, large population-based data are particularly valuable to assess relatively rare outcomes like hospital admissions in pediatric type 1 diabetes. Another strength of the present analysis is that we did not exclude patients with therapy switch, contrary to precedent studies [5]. Thus, individual variations in pump and injection use with different durations were taken into account, reflecting the real-world complexity.

Nevertheless, this analysis has limitations. In the DPV registry, parents’ education level is incompletely documented and household income is not available. We assume that such socio-economic baseline characteristics, which are known to have an impact on the access to diabetes technology [21], are not equally distributed between the patients using pump therapy and those using injection therapy. To address this limitation, we performed the analysis in more than 13,000 patients with one or many therapy switches and report similar results in these patients who used both therapy modalities. Moreover, we performed multiple regression analysis in order to adjust for age group, sex, and migration in the period of diagnosis, and used stratification methods for the analysis during the course of the disease. Overall, our results were robust.

Due to historical reasons and funding of diabetes care, some countries still have high hospitalization rates in pediatric diabetes. In the near future, patient access to advanced technology, like automated insulin delivery (close loop) systems, is anticipated to improve [22], and thus, the importance of diabetes technology education will increase. By consideration of the high costs related to inpatient education and training, the diabetes community should continue to discuss opportunities of promoting outpatient approaches [23, 24]. Nevertheless, the present study underlines that, even in countries with high hospitalization rates and hospitalizations at diagnosis of longer duration, the use of pump therapy is associated with a lower number of hospital days in the long-term, especially for children under 5 years of age.

## Data Availability

M.A. is the guarantor of this work and, as such, had full access to all the data in the study, and takes responsibility for the integrity of the data and the accuracy of the data analysis.

## Abbreviations

BMI: Body mass index
CI: Confidence interval
DKA: Diabetic ketoacidosis
DPV: German Diabetes Prospective Follow-up Registry
LMS method: Method using lambda-mu-sigma parameters
PY: Person-year
SDS: Standard deviation score
